# Overexpression of CDCP1 is Associated with Poor Prognosis and Enhanced Immune Checkpoints Expressions in Breast Cancer

**DOI:** 10.1155/2022/1469354

**Published:** 2022-08-31

**Authors:** Jinlu Zhao, Jie Mei, Fengxu Wang, Xinyuan Zhao, Yi Ren, Xingyu Zhao, Wang Li, Erli Gao

**Affiliations:** ^1^Department of General Surgery, Τhe Fourth Affiliated Hospital of Harbin Medical University, Harbin 150001, China; ^2^Department of Oncology, The Affiliated Wuxi People's Hospital of Nanjing Medical University, Wuxi 214023, China; ^3^Wuxi College of Clinical Medicine, Nanjing Medical University, Wuxi 214023, China; ^4^Department of Occupational Medicine and Environmental Toxicology, Nantong Key Laboratory of Environmental Toxicology, School of Public Health, Nantong University, Nantong 226019, China; ^5^Department of General Surgery, The First Affiliated Hospital of Soochow University, Suzhou 215006, China

## Abstract

CUB-domain containing protein 1 (CDCP1) is a transmembrane protein acting as an effector of SRC family kinases, which play an oncogenic role in multiple human cancers. However, its clinical and immune correlations in breast cancer (BrCa) have not been explored. To define the expression, prognostic value, and potential molecular role of CDCP1 in BrCa, multiple public datasets, and an in-house cohort were used. Compared with paratumor tissue, CDCP1 was remarkably upregulated in the tumor tissues at both mRNA and protein levels. In the in-house cohort, CDCP1 protein expression was related to several clinicopathological parameters, including age, ER status, PR status, molecular type, and survival status. Kaplan–Meier analysis and Cox regression analysis exhibited that CDCP1 was an important prognostic biomarker in BrCa. In addition, enrichment analysis uncovered that CDCP1 was not only involved in multiple oncogenic pathways, but correlated with overexpression of immune checkpoints. Overall, we reported that increased expression of CDCP1 is a favorable prognostic factor in patients with BrCa. In addition, the correlations between CDCP1 and immune checkpoints provide a novel insight into the adjuvant treatment for immune checkpoint blockade via targeting CDCP1.

## 1. Introduction

Breast cancer (BrCa) is a common malignancy with the highest morbidity and terrible mortality among all cancers worldwide [1]. According to the latest statistical data, there will be 290,560 estimated new cases and more than 43,000 estimated deaths in 2022 in the USA [1]. In addition, the morbidity of BrCa has been slowly increasing by approximately 0.5% per year since the mid-2000 s partly due to continued decreases in fertility and increase in excess body weight [2]. Although the prognosis of BrCa has been persistently improved with the rapid development of comprehensive and personalized therapeutic strategies, including chemotherapy, radiotherapy, targeted therapy, and immunotherapy, not all patients could benefit from the established treatment options [3]. Thus, reliable biomarkers are important for the prediction of drug-specific responses and prognosis in BrCa patients.

CUB-domain containing protein 1 (CDCP1) encodes a transmembrane protein that contains three extracellular CUB domains and functions as an effector of SRC family kinases [4]. Previous studies have revealed that CDCP1 is oncogenic in several human cancers *via* regulating tyrosine phosphorylation-dependent cellular functions, and then promotes tumor invasion and metastasis [5, 6]. A growing number of studies uncover the multiple roles of CDCP1 in cancers. CDCP1 is highly expressed in mesenchymal glioma subtypes, which may promote proneural-mesenchymal transformation [7]. Given CDCP1 is highly expressed in RAS-driven cancers, targeting a proteolytic neoepitope on CDCP1 is a pan-cancer approach to control RAS-driven cancers [8]. In addition, CDCP1 is a prognostic biomarker in early non-small-cell lung cancer, and its high expression predicts a poor prognosis [9]. Although several studies have preliminarily investigated the oncogenic role of CDCP1 in BrCa [10, 11], systematic analysis based on transcriptomics and its prognostic value in BrCa has not been defined yet.

In the current research, we aimed to investigate the expression, prognostic value, and potential molecular role of CDCP1 in BrCa using multiple public datasets and an in-house cohort. We reported that CDCP1 was remarkably upregulated in BrCa tissues and enriched in the HER2-positive and the triple-negative subtypes. In addition, high expression of CDCP1 predicted poor prognosis in BrCa. Moreover, we also performed a systematic analysis of CDCP1 using the transcriptomic data and found that CDCP1 was not only involved in multiple oncogenic pathways but correlated with overexpression of immune checkpoints. Overall, we systematically analyzed the role of CDCP1 and emphasized the remarkable correlation between CDCP1 and immune checkpoints in BrCa.

## 2. Materials and Methods

### 2.1. UALCAN Database Analysis

UALCAN (https://ualcan.path.uab.edu/) is an online open-access platform using omics data and clinical information from The Cancer Genome Atlas (TCGA) and the Clinical Proteomic Tumor Analysis Consortium (CPTAC) databases [12]. It could be utilized to analyze transcriptional and protein levels of potential genes of interest between tumor and paratumor tissues and their association with clinicopathologic features. In the current study, the UALCAN tool was utilized to analyze the transcriptional and protein levels of CDCP1 in BrCa and paratumor tissues and its association with clinical stages and molecular subclasses. All the BrCa cases available in the TCGA and the CPTAC subdatabases were included in our study.

### 2.2. Kaplan-Meier Plotter Database Analysis

Kaplan-Meier plotter (https://kmplot.com/analysis/) is a web-based tool integrating gene expression cohorts, clinical information, and survival data [13]. All cancer samples accessible on the Kaplan–Meier plotter were utilized to assess the prognostic values of CDCP1 in BrCa. The mean expression of probe sets (1554110_at and 218451_at) was used to estimate the CDCP1 expression. BrCa patients were divided into the low- and high-CDCP1 expression groups according to the median level of CDCP1, with the rest of the settings set to default. Kaplan–Meier survival plots were derived to display all of the cohorts. The log-rank*P* value, 95 percent confidence interval (95%CI), and hazard ratio (HR) were computed and shown online.

### 2.3. Correlation Genes Screen and Enrichment Analysis

Linked Omics (https://www.linkedomics.org/login.php) is a web-based tool used to handle the TCGA data [14]. In this research, the Linked Omics was used to screen genes that correlated with CDCP1 in BrCa. Genes with a correlation coefficient ≥ 0.2 or ≤ −0.2 were deemed to be candidates. For all parameters, the default choices were utilized. To identify the CDCP1-related biological functions and pathways, all correlated genes were used for enrichment analysis. We downloaded the h.all.v7.4.symbols.gmt and c2.cp.wikipathways.v7.4.symbols.gmt subclasses from the molecular signatures database [15], which were used as the background. The enrichment analysis was conducted using the R package “clusterProfiler.” To obtain the results of gene set enrichment, the minimum gene set was set to 5 and the maximum gene was set to 5000. The top 5 terms were exhibited in this research.

### 2.4. Estimation of the Immunological Characteristics of the TME

The RNA-sequencing (RNA-seq) data of BrCa in the TCGA database was obtained from the UCSC Xena (https://xenabrowser.net/datapages/). The public data was utilized to investigate the immunological features. First, the ESTIMATE algorithm was conducted to estimate tumor purity, ESTIMATE score, immune score, and stromal score [16], and their correlations with CDCP1 expression were next assessed. Next, several gene markers related to the tumor microenvironment (TME) as well as immune checkpoints were obtained from a previous publication [17] and their correlations with CDCP1 expression were evaluated. Furthermore, the correlations between CDCP1 expression and 150 immune-related genes, including chemokines, receptors, MHC molecules, immunoinhibitors, and immunostimulators, were assessed. In addition, the CIBERSOR method [18] was used to estimate the abundance of tumor-infiltrating immune cells (TIICs) based on gene expression profiles using the R package IOBR, and the correlations between CDCP1 expression and TIICs abundance were also evaluated.

### 2.5. Collection of BrCa Specimens

The BrCa (Cat. HBre-Duc159Sur-01) tumor tissue microarray (TMA) was purchased from Outdo BioTech (Shanghai, China). A total of 119 tumor samples and 40 paired paratumor samples were contained in this research. Detailed clinic-pathological and follow-up data were provided by Outdo BioTech. Ethical approval was granted by the Clinical Research Ethics Committee in Outdo Biotech (Shanghai, China).

### 2.6. IHC Staining and Semiquantitative Assessment

Immunohistochemistry (IHC) staining was conducted on the above sections according to the standardized procedures. The primary antibodies used were as follows: anti-CDCP1 (1 : 500 dilution, Cat. AP73474, Abcepta) and anti-EGFR (Ready-to-use, Cat. PA135, Abcarta). Antibody staining was visualized with DAB and hematoxylin counterstain, and stained sections were captured using Aperio Digital Pathology Slide Scanners. The stained sections were independently evaluated by two pathologists. Expression levels of CDCP1 and EGFR in tumor cells were semiquantitatively assessed by estimating the immunoreactivity score (IRS) [19]. Briefly, the percentage of positively stained cells was scored as 0–4: 0 (< 5%), 1 (6–25%), 2 (26–50%), 3 (51–75%) and 4 (> 75%). The staining intensity was scored as 0–3: 0 (negative), 1 (weak), 2 (moderate), and 3 (strong). The IRS equals the percentages of positive cells multiplied with staining intensity.

### 2.7. Acquisition of GSE173839 Dataset

The GSE173839 dataset included RNA-seq data of BrCa from 71 patients on the durvalumab/olaparib arm, which were downloaded from the Gene Expression Omnibus (https://www.ncbi.nlm.nih.gov/geo/) [20]. We extracted the expression data of CDCP1 and PD-L1, explored the predictive value of CDCP1 for immunotherapy, and compared its predictive value with PD-L1.

### 2.8. Statistical Analysis

All statistical analyses were conducted using SPSS 26.0 and R 4.0.2. All data are presented as means ± SDs. The difference between the two groups was analyzed by Student's *t*-test or Mann–Whitney test. Survival analysis was performed by log-rank test and Cox regression analysis. Associations between CDCP1 expression and clinic-pathological features were assessed using the chi-square test or corrected chi-square test. Correlation analysis between two variables was analyzed by the Pearson test. All statistical tests were two-sided, and *P* value ≤ 0.05 was considered statistically significant.

## 3. Results

### 3.1. CDCP1 was Upregulated in BrCa Tissues

**Figure 1:**
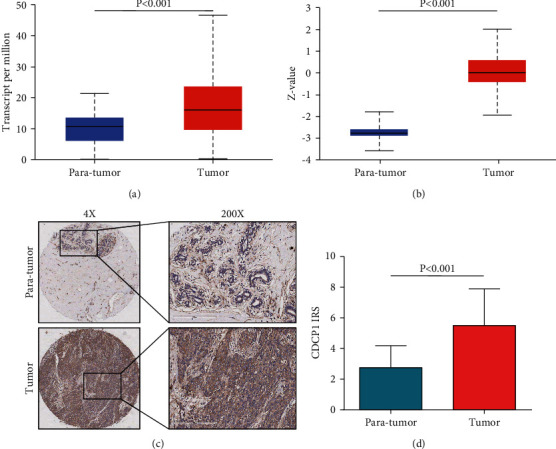
Expression of CDCP1 in paratumor and BrCa tissues. (a) Comparison of CDCP1 mRNA expression in paratumor and BrCa tissues in the TCGA dataset based on data mining via UALCAN. (b) Comparison of CDCP1 protein expression in paratumor and BrCa tissues in the CPTAC dataset based on data mining via UALCAN. (c) Representative images revealing CDCP1 expression in tumor and paratumor tissues using anti-CDCP1 staining. (d) Expression levels of CDCP1 in tumor and paratumor tissues in the in-house cohort.

First, we compared the expression levels of CDCP1 in tumor and paratumor samples using the TCGA, the CPTAC, and the in-house cohorts. In the TCGA cohort, the transcriptional level of CDCP1 was notably upregulated in BrCa tissues (Figure 1(a)). In addition, CDCP1 protein was also overexpressed in tumor samples in the CPTAC cohort (Figure 1(b)). Moreover, we utilized the IHC staining to detect CDCP1 expression BrCa and paratumor tissues, and the results showed that CDCP1 protein was significantly enhanced in tumor samples (Figure 1(c)–1(d)). Overall, CDCP1 was highly expressed in BrCa tissues, which could participate in the oncogenesis of BrCa.

### 3.2. CDCP1 Was Related to the Molecular Type of BrCa

**Figure 2:**
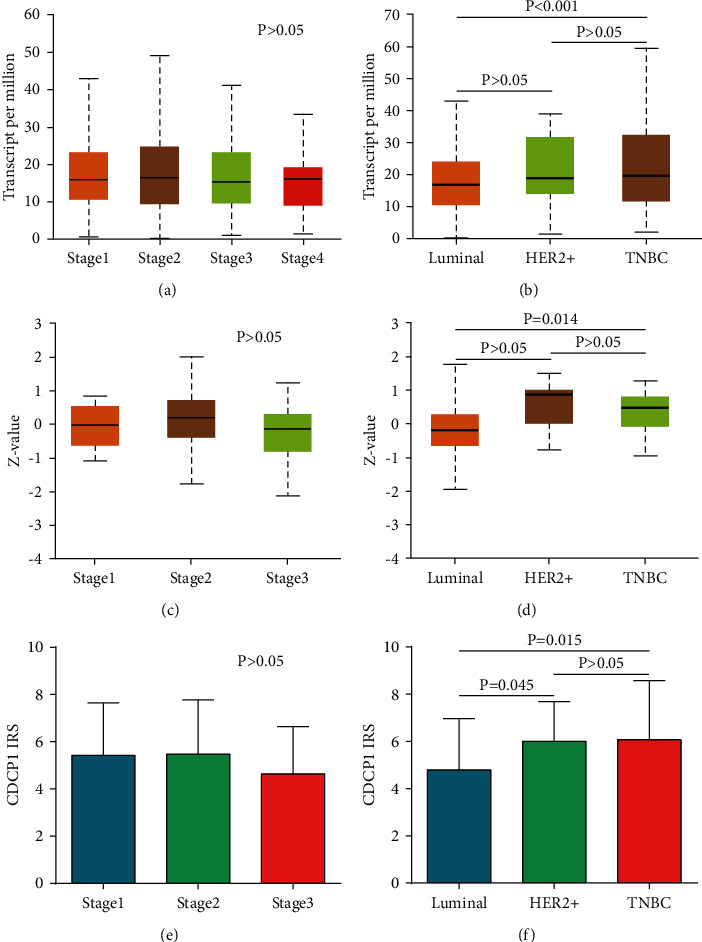
Expression of CDCP1 in BrCa tissues with various stages and subtypes. (a, b) Comparison of CDCP1 mRNA expression in BrCa tissues with various stages and subtypes in the TCGA dataset based on data mining via UALCAN. (c, d) Comparison of CDCP1 protein expression in BrCa tissues with various stages and subtypes in the CPTAC dataset based on data mining via UALCAN. (e, f) Comparison of CDCP1 protein expression in BrCa tissues with various stages and subtypes in the in-house cohort.

Next, the associations between CDCP1 protein expression and clinicopathological features in BrCa were evaluated in the in-house cohort. As shown in Table 1, the expression of CDCP1 was not related to tumor differentiation, T stage, AJCC stage, and HER2 status. However, CDCP1 was significantly associated with age, N stage, ER status, PR status, molecular type, and survival status. We also compared the expression levels of CDCP1 in different TNM stages and molecular subtypes in the TCGA, the CPTAC, and the in-house cohorts. The results exhibited that CDCP1 was not varied in tumor tissues with different TNM stages (Figures 2(a), 2(c), 2(e)), but upregulated in HER-positive and triple-negative subtypes (Figures 2(b), 2(d), 2(f)). Taken together, the expression of CDCP1 was associated with the molecular type of BrCa.

### 3.3. Overexpression of CDCP1 Predicted Poor Prognosis of BrCa

**Figure 3:**
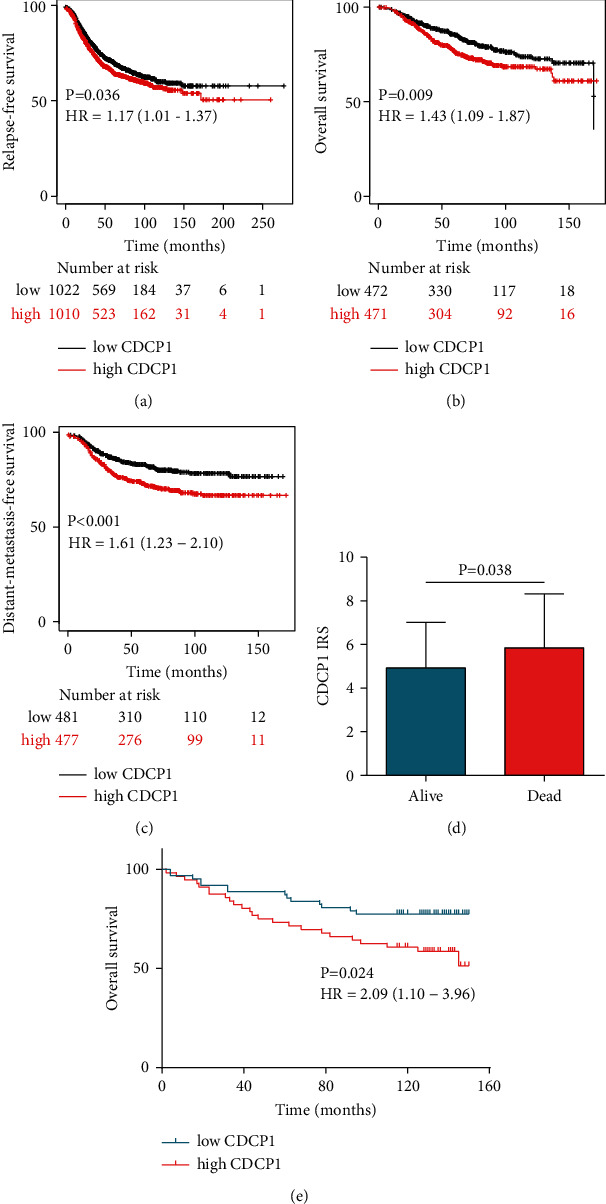
Prognostic value of CDCP1 in BrCa patients. (a, b, c) RFS, OS, and DMFS curves were plotted to assess the prognostic value of CDCP1 mRNA expression in BrCa using the Kaplan–Meier Plotter database. (d) Expression levels of CDCP1 in tumor tissues from alive and dead patients. (e) OS curves were plotted to evaluate the prognostic value of CDCP1 protein expression in the in-house cohort.

Given the notable association between CDCP1 expression and survival status, we subsequently investigated the prognostic value of CDCP1 in BrCa. In the Kaplan–Meier plotter database, high transcriptional expression of CDCP1 was remarkably associated with poor relapse-free survival (RFS), overall survival (OS), and distant-metastasis-free survival (DMFS) (Figures 3(a)–3(c)). In addition, in the in-house cohort, CDCP1 was upregulated in the tumor tissues of patients who died during the follow-up processes (Figure 3(d)). Similarly, high expression of CDCP1 protein expression predicted poor OS in the in-house cohort (Figure 3(e)). Furthermore, both univariate and multivariate Cox regression analyses revealed that high expression of CDCP1 was an independent prognostic factor in BrCa patients (Table 2). Collectively, CDCP1 was a significant prognostic biomarker in BrCa.

### 3.4. Analysis of CDCP1-Related Potential Functions in BrCa

**Figure 4:**
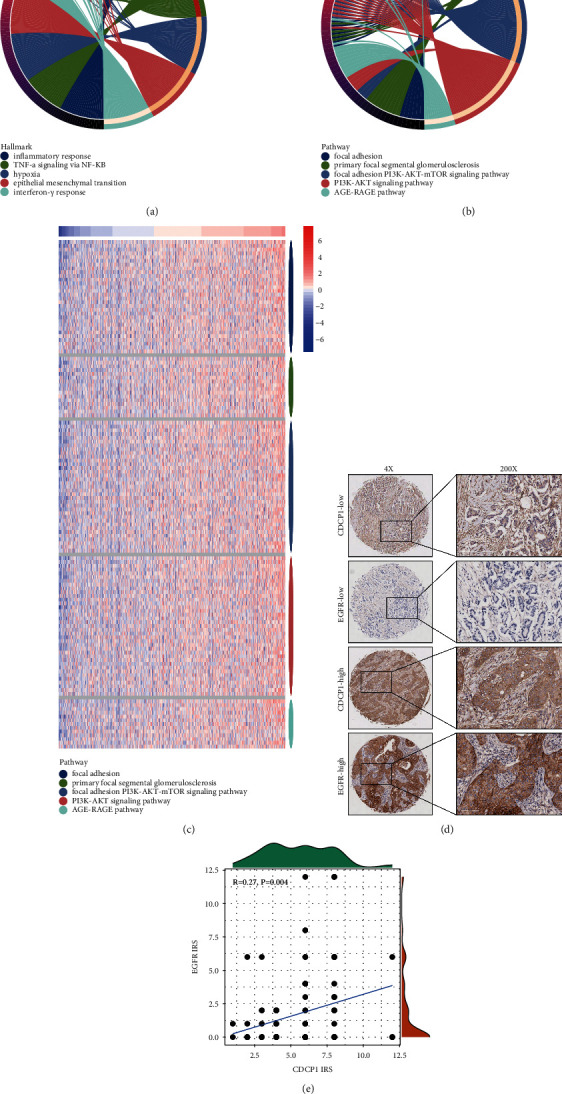
Enrichment analysis of PCGs of CDCP1 in BrCa. (a) Hallmark enrichment analysis of PCGs of CDCP1. (b) Wikipathways enrichment analysis of PCGs of CDCP1. (c) Heatmap showing PCGs expressions in the Wikipathways enrichment analysis. (d) Representative images revealing CDCP1 and EGFR expressions in tumor tissues using anti-CDCP1 and anti-EGFR staining. (e) Correlation between CDCP1 and EGFR in BrCa tissues in the in-house cohort.

Subsequently, we tried to investigate CDCP1-related functions in BrCa. Genes correlated with CDCP1 in BrCa with correlation coefficient ≥0.2 or ≤ −0.2 were deemed to be candidates (Figures S1A–S1C). Then, hallmark and Wikipathways gene set analyses of positively correlated genes (PCGs) and negatively correlated genes (NCGs) were conducted, respectively. PCGs mainly participated in an inflammatory response, TNF-*α* signaling, hypoxia, epithelial-mesenchymal transition (EMT), and interferon-*γ* response (Figure 4(a)), and was involved in focal adhesion, primary focal segmental glomerulosclerosis, PI3K-AKT signaling pathway, and AGE-RAGE pathway (Figure 4(b)). The enrichment results of Wikipathways were visualized in Figure 4(c). Given that EGFR was as a significant gene that positively correlated with CDCP1, we validated the correlation between these genes in the in-house cohort, and the result exhibited that CDCP1 was significantly correlated with EGFR (Figures 4(d)–4(e)). In addition, the enrichment results of NCGs were scattered, which were exhibited in Figure S2. To sum up, CDCP1 may be related to inflammatory and immune responses via regulating multiple pathways in BrCa.

### 3.5. CDCP1 Was Correlated with Immune Checkpoints Expressions in BrCa

**Figure 5:**
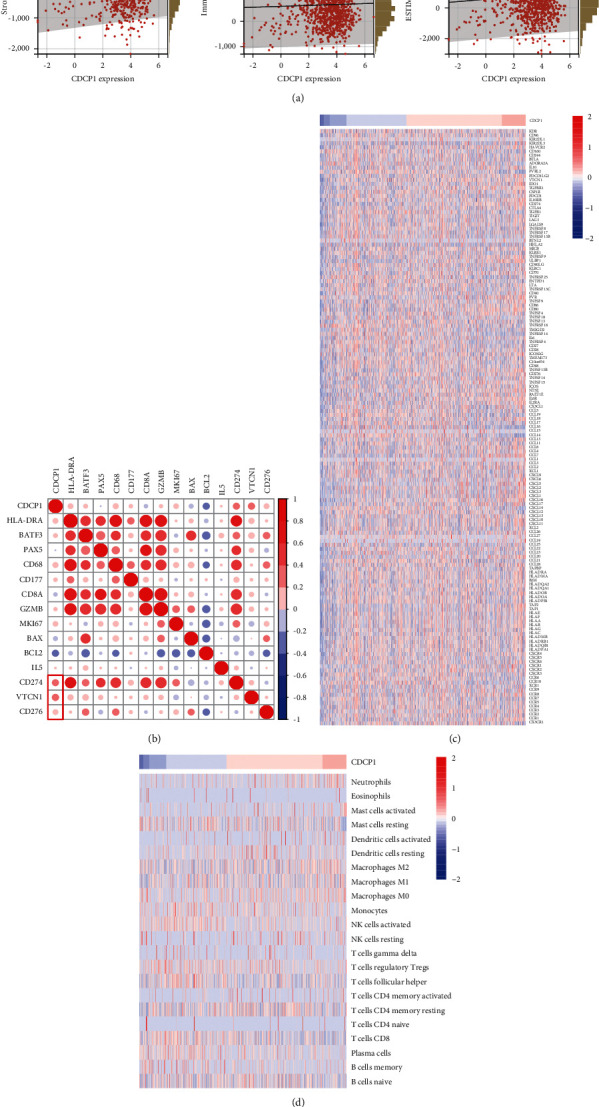
Association between CDCP1 and the immune microenvironment in BrCa. (a) Correlations between CDCP1 and stromal score, immune score, and ESTIMATE score are estimated by the ESTIMATE method. (b) Correlations between CDCP1 and indicated gene expressions. (c) Heatmap showing immunomodulators expressions in BrCa tissues. (d) Heatmap showing TIICs abundance estimated by the CIBERSOR method in BrCa tissues.

**Figure 6:**
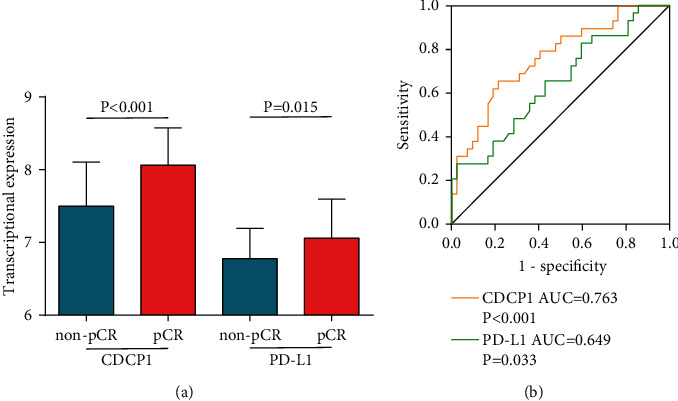
Predictive value of CDCP1 for immunotherapy in BrCa cohort. (a) Expression levels of CDCP1 and PD-L1 in BrCa with various immunotherapeutic responses. (b) Comparison of predictive values of CDCP1 and PD-L1 in BrCa cohort.

Considering the potential relationship between CDCP1 and inflammatory and immune response in BrCa, we next explored the correlations between CDCP1 and gene markers of immune-related events. CDCP1 showed no significant correlation with the stromal score, immune score, and ESTIMATE score (Figure 5(a)). In addition, CDCP1 was also not correlated with MHC molecules, gene markers of multiple immune cells, but positively related to immune checkpoint expressions, including CD274 (PD-L1), CD276 (B7–H3), and VTCN1 (B7–H4) (Figure 5(b)). In addition, a larger throughput analysis showed that CDCP1 was not significantly associated with immune-related genes and TIICs abundance (Figures 5(c)–5(d)). Since CDCP1 was positively correlated with PD-L1, we also examined whether CDCP1 could be a biomarker for immunotherapy in BrCa. The results showed that CDCP1 and PD-L1 were highly expressed in BrCa tissues with a good response (Figure 6(a)), and the predictive value of CDCP1 was even higher than PD-L1 in the GSE173839 dataset (Figure 6(b)). Overall, CDCP1 was related to enhanced immune checkpoint expressions and could predict the response to immunotherapy in BrCa.

## 4. Discussion

CDCP1 has been revealed to be significantly dysregulated in tumor tissues and accelerates progression in several malignancies [21]. CDCP1 is eminently located on the cytomembrane, which lies at the nexus of critical tumorigenic signaling cascades, containing the SRC-PKC*δ*, PI3K-AKT, WNT, and RAS-ERK axes, the oxidative pentose phosphate pathway, and fatty acid oxidation, making significantly functional contributions to tumor progression and development [21]. In addition, CDCP1 has a notable prognostic role in cancer. Ikeda et al. performed a multivariate Cox regression analysis of 200 lung adenocarcinoma patients and revealed that high-CDCP1 expression was an independent prognostic factor for OS in lung adenocarcinoma [22]. Dagnino et al. suggested that the circulating serum level of CDCP1 was related to the risk of developing lung cancer, especially in patients with tobacco exposure [23]. However, a systematic analysis of CDCP1 in BrCa has not been performed yet.

In this research, we reported that CDCP1 was significantly overexpressed in BrCa tissues and highly expressed in the HER2-positive and triple-negative subtypes. Previous research has revealed that CDCP1 is a novel marker of triple-negative breast cancer [24] and promotes tumor progression via reduction of lipid-droplet abundance and stimulation of fatty acid oxidation [25]. In addition, CDCP1 could interact with HER2 and enhance HER2-driven tumorigenesis in BrCa [26]. Thus, the enrichment of CDCP1 might be crucial for the aggressiveness of the HER2-positive subtype. Furthermore, high expression of CDCP1 predicted poor prognosis in BrCa, which could be a novel biomarker for prognostic assessment in BrCa. Moreover, we also performed a systematic analysis of CDCP1 using the transcriptomic data and found that CDCP1 was not only involved in multiple oncogenic pathways, but correlated with overexpression of immune checkpoints.

With the rapid development of bioinformatics-assisted tumor immunity studies, immuno-correlations analysis has been emerging as a hotspot in the field of cancer research. A growing number of novel immune biomarkers has been identified [27–29]. Most immune biomarkers in the tumor were correlated with the inflamed immune microenvironment, such as enhanced chemokines, MHC molecules, and effective TIICs, and also correlated with immune checkpoint expressions [30, 31]. In the current research, we found that CDCP1 was not related to the inflamed immune microenvironment, but positively correlated with immune checkpoint expressions, including CD274 (PD-L1), CD276 (B7–H3), and VTCN1 (B7–H4). Thus, CDCP1 might be a crucial regulator that contributed to immune evasion via promoting immune checkpoint expressions.

It has been reported that CDCP1 is crucial for the activation of RAS in cancer [8], and participates in multiple oncogenic pathways, such as EGF signaling [32] and HGF signaling [33]. In addition, we predicted that CDCP1 was involved in TNF-*α* signaling, hypoxia, EMT, interferon-*γ* response, PI3K-AKT signaling, and AGE-RAGE signaling. Most of these pathways are associated with the regulation of immune checkpoints in cancer. For example, PD-L1 could be upregulated in ZEB1 and miR-200 dependent manners EMT-activated human breast cancer cells [34]. In addition, immune checkpoint molecules PD-L1 and B7–H3 were notably upregulated during TGF-*β*1-induced EMT [35]. Although our current study suggested potential relationships of CDCP1 to these pathways, the lack of confirmation from the molecular biology level remained an unavoidable shortcoming of this study.

## 5. Conclusion

In conclusion, we revealed that CDCP1 was highly expressed in BrCa tissues and enriched in the HER2-positive and triple-negative subtypes, which also functioned as a novel prognostic biomarker in BrCa. In addition, CDCP1 was positively correlated with immune checkpoint expressions in BrCa, and several possibly related pathways were also suggested. Overall, we systematically investigated the role of CDCP1 in BrCa and provided a possible insight into the CDCP1-mediated overexpression of immune checkpoints.

## Figures and Tables

**Table 1:** Associations between CDCP1 expression status and clinic-pathological characteristics in BrCa.

Clinic-pathological characteristics		Case	CDCP1 expression		*χ* ^2^ value	*P* Value
			Low	High		
Age	≤50	58	37	21	5.790	0.016
	>50	60	25	35		
	NA	1				

Differentiation	I (I–II)	40	20	20	0.107	0.744
	II&III	79	42	37		

T stage^∗^	T1	25	10	15	—	0.281
	T2	81	43	38		
	T3	12	8	4		
	NA	1				

N stage^∗^	N0	43	17	26	—	0.044
	N1	36	21	15		
	N2	28	16	12		
	N3	8	7	1		
	NA	4				

TNM stage^∗^	1	7	3	4	—	0.224
	2	69	33	36		
	3	39	25	14		
	NA	4				

ER status	Negative	43	15	28	8.460	0.004
	Positive	75	47	28		
	NA	1				

PR status	Negative	51	18	33	11.367	0.001
	Positive	66	44	22		
	NA	2				

HER2 status	Negative	91	45	46	1.089	0.297
	Positive	28	17	11		

Molecular type	Luminal	78	50	28	12.439	0.002
	HER2+	15	5	10		
	TNBC	25	7	18		
	NA	1				

Survival status	Alive	80	48	32	5.541	0.019
	Dead	38	14	24		
	NA	1				

*Note.* ^∗^ Checked using the corrected chi-square test.

**Table 2:** Univariate and multivariate analysis of survival in patients with BrCa.

Clinic-pathologicalcharacteristics	Univariate analysis			Multivariate analysis		
	HR	95%CI	*P* Value	HR	95%CI	*P* Value
Age	1.21	0.64–2.29	0.559			
Grade	1.33	0.66–2.68	0.429			
T stage	1.54	0.86–2.78	0.149			
N stage	1.36	0.98–1.90	0.067			
TNM stage	2.13	1.19–3.84	0.011	2.90	1.51–5.56	0.001
ER status	0.43	0.22–0.80	0.008	3.19	0.51–20.07	0.217
PR status	0.34	0.18–0.67	0.002	0.55	0.19–1.57	0.260
HER2 status	1.04	0.49–2.19	0.924			
Molecular type	1.84	1.30–2.60	0.001	2.38	0.92–6.17	0.075
CDCP1 expression	2.10	1.08–4.06	0.028	2.13	1.04–4.34	0.038

## Data Availability

All data supporting the results of this study are shown in this published article and supplementary documents. In addition, original omics data for bioinformatics analysis could be obtained from corresponding platforms.

## Supplementary Materials

Supplementary Materials Figure S1. The genes co-expressed with CDCP1 in BrCa. Figure S2. Enrichment analysis of NCGs of CDCP1 in BrCa.
