# Supplementary material for: Overexpression of CDCP1 is Associated with Poor Prognosis and Enhanced Immune Checkpoints Expressions in Breast Cancer
Source: J Oncol. 2022 Aug 31;2022:1469354. doi: 10.1155/2022/1469354 (PMC9452972; doi:10.1155/2022/1469354)
Supplement: Supplementary Materials — Figure S1. The genes co-expressed with CDCP1 in BrCa. Figure S2. Enrichment analysis of NCGs of CDCP1 in BrCa. [file 1469354.f1.docx]

**Supplementary Figures**


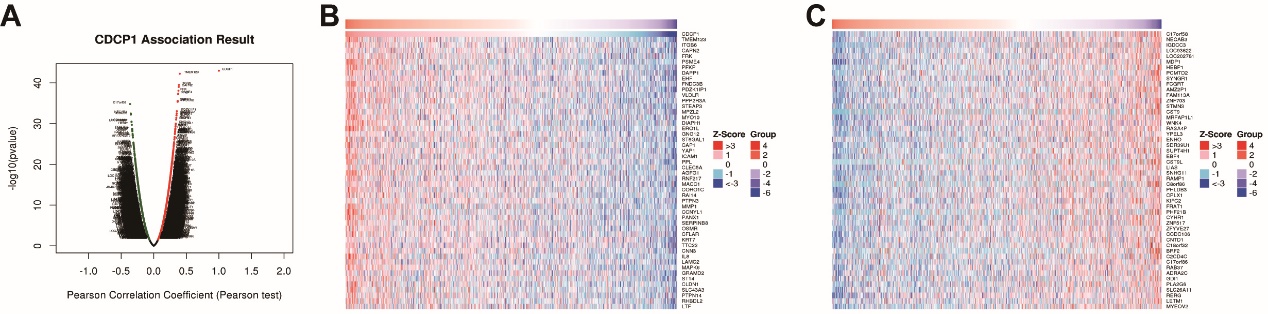


**Figure S1.** **The genes co-expressed with CDCP1 in BrCa.**

(A) The global correlated genes of CDCP1 identified by Pearson test in the TCGA cohort. (B) Heatmap showing top 50 genes positively correlated with CDCP1 in BrCa. (C) Heatmap showing top 50 genes negatively correlated with CDCP1 in BrCa.


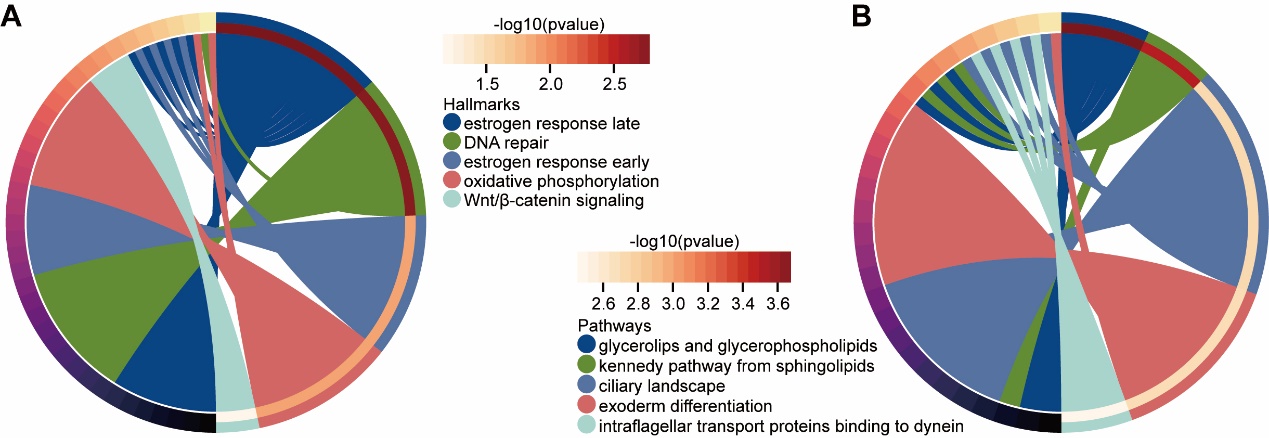


**Figure S2. Enrichment analysis of NCGs of CDCP1 in BrCa.**

(A) Hallmark enrichment analysis of NCGs of CDCP1. (B) Wikipathways enrichment analysis of NCGs of CDCP1.
